# PDZD8 is not the ‘functional ortholog’ of Mmm1, it is a paralog

**DOI:** 10.12688/f1000research.15523.1

**Published:** 2018-07-16

**Authors:** Jeremy G. Wideman, Dario L. Balacco, Tim Fieblinger, Thomas A. Richards

**Affiliations:** 1 Department of Biosciences, University of Exeter, Exeter, EX4 4QD, UK; 2 Wissenschaftskolleg zu Berlin, Berlin, 14193, Germany; 3 School of Life Sciences, University of Nottingham, Nottingham, NG7 2UH, UK; 4 Basal Ganglia Pathophysiology Unit, Department of Experimental Medical Science, Lund University, Lund, 22184, Sweden

**Keywords:** Pdz8, ERMES, paralog, ortholog, evolution

## Abstract

Authors of a recent paper demonstrate that, like ERMES (ER-mitochondria encounter structure) in fungal cells, PDZD8 (PDZ domain containing 8) tethers mitochondria to the ER in mammalian cells. However, identifying PDZD8 as a “functional ortholog” of yeast Mmm1 (maintenance of mitochondrial morphology protein 1) is at odds with the phylogenetic data. PDZD8 and Mmm1 are paralogs, not orthologs, which affects the interpretation of the data with respect to the evolution of ER-mitochondria tethering. Our phylogenetic analyses show that PDZD8 co-occurs with ERMES components in lineages closely related to animals solidifying its identity as a paralog of Mmm1. Additionally, we identify two related paralogs, one specific to flagellated fungi, and one present only in unicellular relatives of animals. These results point to a complex evolutionary history of ER-mitochondria tethering involving multiple gene gains and losses in the lineage leading to animals and fungi.

Hirabayashi *et al.* ^1^ show that PDZD8 (PDZ domain containing 8) is an SMP domain-containing protein involved in ER-mitochondria tethering and regulation of Ca ^2+^ dynamics in mammalian neurons. We do not dispute the authors’ interesting results with respect to PDZD8 function in mammalian cells. However, claims made by the authors regarding the evolutionary relationship of this gene with fungal homologs represents a misuse of the term ortholog, where the term homolog or, more appropriately, paralog is correct. This misuse has consequences for interpreting data in the paper, including in the domain-swapping experiments conducted as part of the complementation assay. The misclassification affects how their data should be interpreted and results in confused explanations of how ER-mitochondria contact sites evolved in animals and fungi. Approaching the data with correct terminology alleviates these problems and illuminates interesting possibilities about trait evolution in fungi and animals.

Orthology and paralogy are special cases of homology ( Figure 1). Orthologous genes arise by speciation events. Mouse α-haemoglobin is orthologous to human α-haemoglobin. Paralogous genes arise by gene duplication events. Duplication of a globin gene in an ancestor of vertebrates gave rise to two haemoglobin families in which α- and β-haemoglobin subsequently evolved. Therefore, α- and β-haemoglobin, regardless of which organisms they appear in, are paralogs. In this simple case, the orthologous proteins perform the same function in different organisms (i.e. they are isofunctional orthologs ^2^). However, orthologs can diverge and perform different functions in different lineages (heterofunctional orthologs). An example of heterofunctional orthologs is animal Miro (mitochondrial rho GTPase), which, in yeast, is called Gem1 (GTPase EF-hand protein of Mitochondria) and has been suggested to interact with ERMES (ER-mitochondria encounter structure) in fungi ^3, 4^. Miro is important for microtubule-dependent mitochondrial motility in animals (reviewed by Reis *et al.* ^5^). However, although a Miro ortholog is present in all eukaryotic lineages, it does not function in microtubule-dependent mitochondrial motility in yeast, *Neurospora*, *Arabidopsis*, or *Dictyostelium* ^5– 7^. Thus, functionality alone does not imply orthology.

**Figure 1.**
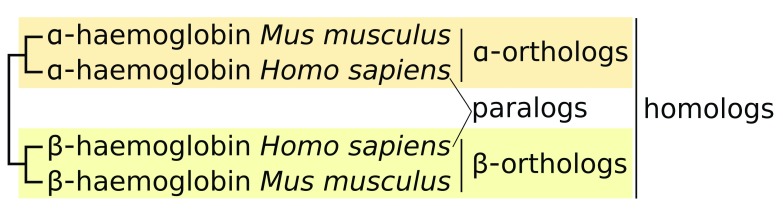
Orthologs versus paralogs: haemoglobin as an example. Orthologs are a consequence of speciation, whereas paralogs are a consequence of gene duplication. Human α- and β-haemoglobin share 43% identity whereas Human α-haemoglobin and Mouse α-haemoglobin share 87% identity. When performing phylogenetic analyses, the orthologous α-haemoglobin subunits from different animals branch together separate from their paralogs, the β-haemoglobin subunits. Taken together, all haemoglobin subunits are homologs.

Any SMP domain is homologous to any other SMP domain because they have shared ancestry, but only those arising via speciation are orthologs. To demonstrate that PDZD8 and Mmm1 (maintenance of mitochondrial morphology protein 1) are “functional orthologs”, Hirabayashi *et al.* ^1^ swap the SMP domain from PDZD8 into the Mmm1 protein in *Saccharomyces cerevisiae* thereby rescuing the defects imparted by loss of Mmm1. But, they also successfully rescue Mmm1 defects by swapping the SMP domain from its paralog, *S. cerevisiae* Mdm12 (mitochondrial distribution and morphology protein 12), into the *S. cerevisiae* Mmm1 protein. The domain swapping experiments should not be interpreted to mean that the proteins (or even the domains) carry out the same function. Instead, these experiments suggest that the paralogous SMP domains from PDZD8, Mmm1, and Mdm12 are biochemically isofunctional in this specific scenario (i.e. the SMP domains can carry out similar functions when they are placed very specifically into the *S. cerevisiae* Mmm1 protein). However, it must be stressed that this does not mean that the proteins themselves are isofunctional. The fact that mammalian PDZD8 and similar proteins from other animals have long C-terminal extensions containing accessory domains (e.g. a PDZ domain and cysteine-rich C1 domains), while Mmm1 and Mdm12 do not, suggests these proteins have different or additional mechanisms of function. Thus, we cannot say that the full proteins are isofunctional homologs and especially not isofunctional orthologs.

The fact that PDZD8 is a homolog of SMP domain-containing proteins like Mmm1 has been demonstrated previously ^8– 11^; but it has also been demonstrated that PDZD8 is not an ortholog of ERMES components ( Figure 2, also see Wideman *et al.* ^11^). This can be seen very clearly in the SMP-domain proteins of *Capsaspora owczarzaki*, which include orthologs of all the ER-mitochondrial contact site SMP proteins (PDZD8, Mmm1, Mdm12, Mdm34). This organism is closely related to animals ^12^, but still retains both a complete ERMES complex and PDZD8 and represents a future model for investigating their differential functions. Interpreting the work of Hirabayashi *et al.* ^1^ from a comparative perspective demonstrates that mitochondria-ER tethering is a function that is conserved deep within this gene family predating the duplication that gave rise to PDZD8 and Mmm1, and indeed, all known ERMES paralogs. This is important because it allows us to phrase the next questions, ‘how have different paralogs expanded, or changed, in function? (is there evidence of heterofunctionality?)’ and ‘why have some evolutionary lineages maintained multiple paralogs while others have tolerated loss?’. When uninformative terms like ‘functional ortholog’ are used, especially in exceptional papers like Hirabayashi *et al.* ^1^, biologists interested in explaining functional differences between organisms are being misled. We stress that only comparative evolutionary methods can identify the starting data for phrasing the above questions and functional data cannot be used when inferring orthology or paralogy. Once questions of orthology and paralogy have been resolved, questions of functional conservation and divergence can be addressed at the bench.

**Figure 2.**
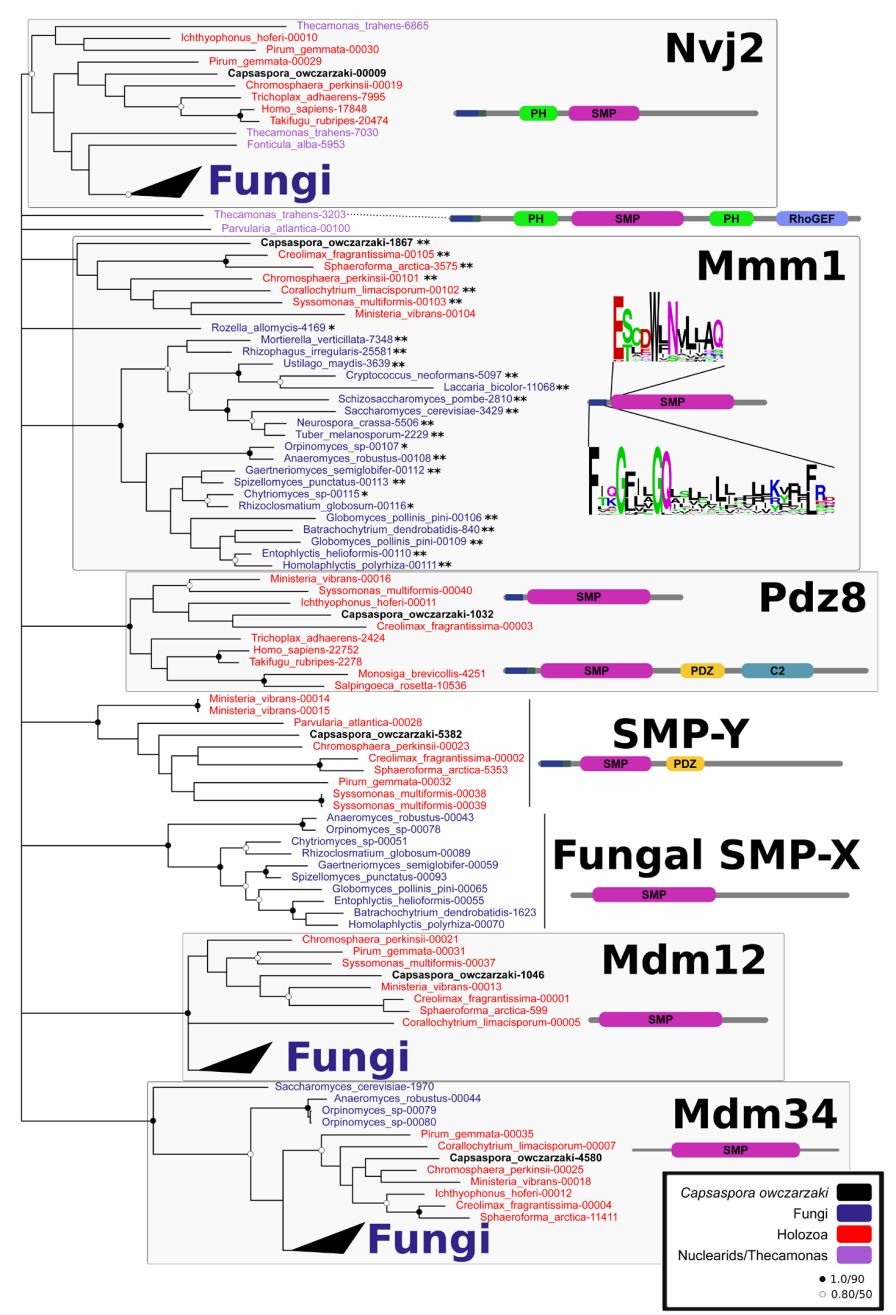
Phylogenetic tree and schematic domain organization of SMP domain-containing proteins. PDZD8 groups to the exclusion of Mmm1, Mdm12, Mdm34, Nvj2 and two unnamed paralogs. SMP domain-containing proteins were gathered from diverse opisthokonts (i.e. animals, fungi, and closely related protists) and their sister species *Thecamonas trahens* (sequences were obtained from public databases (Joint Genome Institute and NCBI) as well as from recently available genomes and transcriptomes ^13, 14^. The SMP domains were aligned and subjected to phylogenetic reconstruction using RaxML v8.2 (100 pseudoreplicates using the LG model) and MrBayes v3.2 (1 million generations using the WAG model) as in 11. Sequences and alignments are available at https://github.com/mbzdlb/PDZD8. Six strongly supported paralogs, including PDZD8, Nvj2, Mdm12, Mdm34, and two unnamed paralogs comprising sequences from flagellated fungi and opisthokont protists, were recovered. Fungal Mmm1 is recovered in a strongly supported clade whereas some proteins designated as Mmm1 previously ^11^ do not. However, sequence inspection identified motifs outside the SMP domains present in both fungal and non-fungal Mmm1s, strongly suggesting that proteins designated as Mmm1 are orthologous. Mmm1 proteins lacking these motifs may represent truncated or mispredicted proteins. Similarly, some predicted Pdz8 proteins lack Pdz domains and C-terminal extensions. Human PDZD8 is considered paralogous to Mmm1 because it groups separately and can be found in organisms that already contain Mmm1-like proteins (e.g. *Capsaspora owczarzaki*). Motifs were visualized using WebLogo ^15^. One asterisk indicates the presence of Mmm1-specific short motif, two asterisks indicate both the short and the transmembrane motifs are present. Support values are as iconized in inset key (MrBayes/RAxML).

## Data availability

All sequences were downloaded from publicly available databases (e.g. Joint Genome Institute or NCBI) or obtained from published transcriptomic and genomic data ^13, 14^. The sequences used to generate Figure 2 are downloadable at https://github.com/mbzdlb/PDZD8.

## Supplementary material

**Supplementary File 1. List of organisms and accession numbers (with database) used to generate Figure 2.** The corresponding sequences can be found at https://github.com/mbzdlb/PDZD8 or from the indicated online database.
