# Supplementary material for: PDZD8 is not the ‘functional ortholog’ of Mmm1, it is a paralog
Source: F1000Res. 2018 Jul 16;7:1088. [Version 1] doi: 10.12688/f1000research.15523.1 (PMC6069729; doi:10.12688/f1000research.15523.1)
Supplement: Peer review history

# Peer review: Referee response for version 1

**DOI:** 10.5256/f1000research.16928.r36318

**Published:** 2018-07-31

**Authors:** Vikram Alva

**Affiliations:** 1 Protein Bioinformatics, Department of Protein Evolution, Max Planck Institute for Developmental Biology, Tübingen, Germany

In this correspondence, Wideman *et al.* comment on a recent seminal study by Hirabayashi *et al.*, which describes the biochemical characterization of the SMP domain-containing protein PDZD8, as an ER-mitochondria tether important for the regulation of Ca ^2+^ dynamics in mammalian neurons. Although the functional data presented by Hirabayashi *et al.* is sound and of great interest, their interpretation of the homologous relationship between PDZD8 and the yeast SMP domain-containing protein Mmm1 is incorrect. Mmm1 is part of the ERMES complex, which tethers the ER to mitochondria in yeast and is important for the exchange of phospholipids between these organelles. This complex contains two further proteins of the SMP family, Mdm12 and Mdm34. Wideman *et al.* ^1^ and we ^2^ have previously shown that some eukaryotes contain all three of these ERMES proteins as well as PDZD8, indicating that they are paralogs. Hirabayashi *et al.*, however, misinterpret PDZD8 to be a functional ortholog of yeast Mmm1, and substantiate this relationship experimentally by substituting the SMP domain of yeast Mmm1 with that of PDZD8 to rescue the phenotypic defects caused by the deletion of Mmm1. Here, Wideman *et al.* present convincing data to show that PDZD8 is a paralog of Mmm1 and not its ortholog. The arguments presented by Wideman *et al.* are sound and will help to clear the confusion regarding the evolutionary relationship between these proteins.

 Minor comment:

 The human PDZD8 protein contains a C1 domain as well as a coiled-coil segment at its C-terminal end. Do other orthologs also contain these domains? If so, the domain composition of PDZD8 shown in Fig. 2 should be updated to show this. Also, HHpred searches suggest that the PDZ domain in human PDZD8 is inserted into the C2 domain; is this the case for other orthologs too?

I have read this submission. I believe that I have an appropriate level of expertise to confirm that it is of an acceptable scientific standard.

## References

[1] WidemanJGGawrylukRMGrayMWDacksJB: The ancient and widespread nature of the ER-mitochondria encounter structure. *Mol Biol Evol*.2013;30(9) : 10.1093/molbev/mst120 2044-9 10.1093/molbev/mst120 23813918

[2] AlvaVLupasAN: The TULIP superfamily of eukaryotic lipid-binding proteins as a mediator of lipid sensing and transport. *Biochim Biophys Acta*.2016;1861(8 Pt B) : 10.1016/j.bbalip.2016.01.016 913-923 10.1016/j.bbalip.2016.01.016 26825693

# Peer review: Referee response for version 1

**DOI:** 10.5256/f1000research.16928.r36417

**Published:** 2018-07-30

**Authors:** Tim P. Levine

**Affiliations:** 1 Institute of Ophthalmology, University College London (UCL), London, UK

In this correspondence, Wideman and colleagues show that PDZD8 (also called PDZ-8 or PDZK8) is not an ortholog of the ERMES subunit Mmm1p. This conclusion by Wideman et al. is well founded, and the underlying argument is well explained in the text here.

 The same point of non-orthology has been said before in Wideman et al., 2015, but was then missed in the first paper that focussed on the function of PDZD8 (Hirabayashi et al., 2017 ^1^). There, the SMP domain from mammalian PDZD8 substituted for the function of  the Mmm1p SMP domain in yeast, which was used to support the view that “The SMP domain of PDZD8 is functionally orthologous to the SMP domain found in yeast Mmm1.” However, a definition of orthologue that I accept is “Homologous sequences are orthologous if they are descended from the same ancestral sequence separated by a speciation event” [https://en.wikipedia.org/wiki/Homology_(biology)]. By that definition, I agree with Wideman et al. that bioinformatic studies indicate PDZD8 and all 3 ERMES subunits with SMP domains, including Mmm1p, are paralogs, not orthologs.

 On that basis the function of PDZD8 may have evolved away from that of ERMES at some time, and might also have converged towards it, but we cannot tell by just showing that the activity of one’s SMP domain (most likely to transfer lipid with fairly low specificity) can be substituted by the other’s SMP domain.

 There are several points where the current article might be marginally improved by revision:

 Improve text and diagram describing domains within PDZD8: apart from the SMP and PDZ domains, PDZD8 is described as containing a C1 domain e.g. NCBI human PDZD8 (1154aa): 841-883. Here that domain is missing from the diagram. In addition, a C2 domain is shown in the diagram in an incorrect way, because the C2 domain sequence in PDZD8 is split. ~65 aa lies between the SMP and PDZ domains (320-372), then the rest (695-800) is before the C1 domain. See Figure 1B in Wong et al. 2017 ^2^.

 The text would benefit from a brief analysis of likely branching of the SMP protein family, based on supplementary data from the 2015 paper, possibly plus extra work to place the different Mmm1 groupings and the two new designations (X and Y).

 The  text should explcitily state that the Mmm1s in C. owczarzaki and other holozoa are variant, rooting onto the main tree separately from fungal Mmm1s, despite the shared presence of the 2 accompanying motifs.

 This raises another point. The diagram adds to cladistic analysis of SMP domain sequences alone by looking for accompanying motifs. How can using accompanying motifs be justified, when accompanying domains are excluded from the orthology/paralogy argument? As SMP sequences can be inherited separately from other elements, I do not fully grasp that C. owczarzaki-1867 is included in Mmm1, while C. owczarzaki-5382 is excluded from Pdz8.

 Understanding the figure might be improved by more information in the legend:

- Say what “ **Fungi**” refers to each of the three times it appears in the diagram. Is it always the same set of fungal sequences or a different group in each case? How many?
- Say that the figure is not the same as appears in ref 11 or its supplementary data, but based on the same data set. 
- Say that Tcbs have been excluded
- For “closely related protists” (line 3) say if every protist has been included or a selection has been made (and how)
- Identify falgellated fungi (compared to non-flagellated)
- Add description of one/two asterisks to key, not at bottom of long legend.

 

 Data set on Git Hub:

 I note that some sequences are incomplete. This should be made explicit.

I have read this submission. I believe that I have an appropriate level of expertise to confirm that it is of an acceptable scientific standard.

## References

[1] HirabayashiYKwonSPaekHPerniceWPaulMLeeJErfaniPRaczkowskiAPetreyDPonLPolleuxF: ER-mitochondria tethering by PDZD8 regulates Cadynamics in mammalian neurons. *Science*.2017;358(6363) : 10.1126/science.aan6009 623-630 10.1126/science.aan6009 PMC581899929097544

[2] WongLHLevineTP: Tubular lipid binding proteins (TULIPs) growing everywhere. *Biochim Biophys Acta*.1864(9) : 10.1016/j.bbamcr.2017.05.019 1439-1449 10.1016/j.bbamcr.2017.05.019 28554774PMC5507252
